# Supplementary material for: Interference-Free Measurement of Urinary Angiotensin-Converting Enzyme (ACE) Activity: Diagnostic and Therapeutic Monitoring Implications
Source: Biomedicines. 2025 Oct 16;13(10):2528. doi: 10.3390/biomedicines13102528 (PMC12562155; doi:10.3390/biomedicines13102528)
Supplement: Supplementary file 1 [file biomedicines-13-02528-s001.zip › biomedicines-3880775-supplementary.pdf]

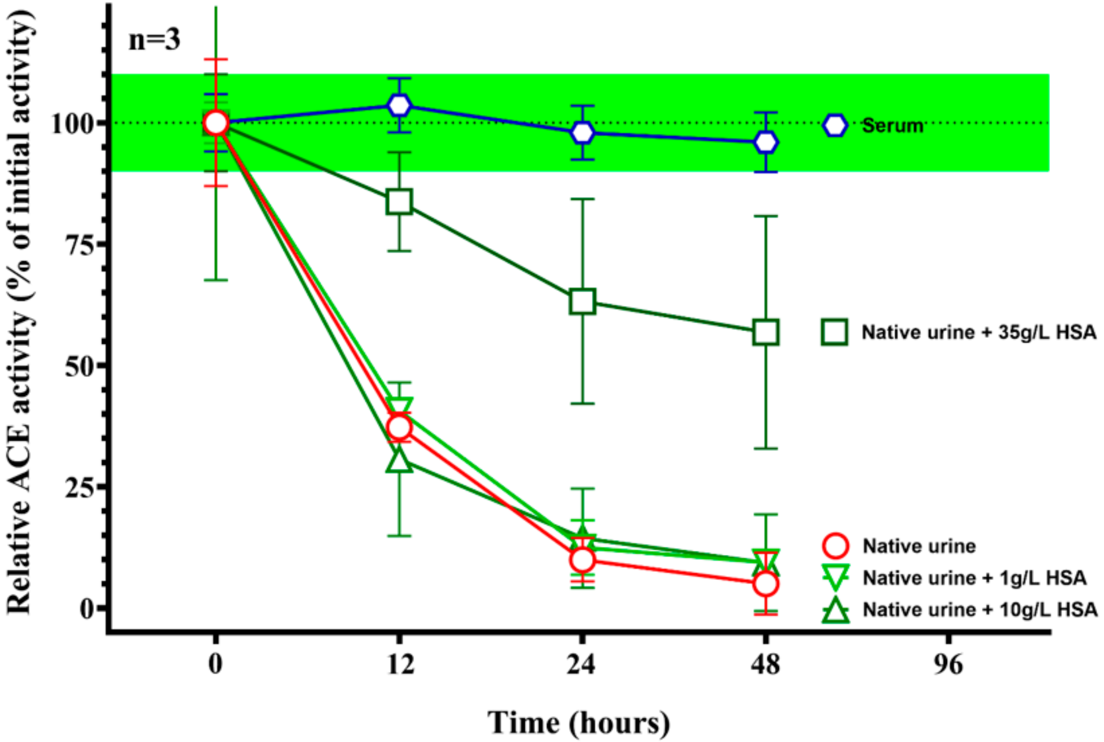

Figure S1. Human serum albumin (HSA) at a final concentration of 35 g/L improved the stability of urinary ACE (uACE) activity during freezing. Native urine (red symbols) showed a marked decline in uACE activity after 24 h of storage at −20 °C. The addition of HSA at final concentrations of 1 g/L (light green symbols) or 10 g/L (dark green symbols) had no appreciable effect on uACE stability. However, supplementation with 35 g/L HSA (green squares) partially preserved uACE activity, although the initial activity level was not fully maintained. In contrast, serum ACE activity (blue symbols) remained stable throughout the tested period. Data represent mean ± SD of three independent experiments, expressed as a percentage of the initial sample activity.
